# Effectiveness of CoronaVac and BNT162b2 COVID-19 mass vaccination in Colombia: A population-based cohort study

**DOI:** 10.1016/j.lana.2022.100296

**Published:** 2022-08

**Authors:** Angel Paternina-Caicedo, Mark Jit, Nelson Alvis-Guzmán, Juan Carlos Fernández, José Hernández, Justo Jesus Paz-Wilches, José Rojas-Suarez, Carmelo Dueñas-Castell, Nelson J. Alvis-Zakzuk, Adrian D. Smith, Fernando De La Hoz-Restrepo

**Affiliations:** a Universidad del Sinú, Cartagena, Colombia; b London School of Hygiene & Tropical Medicine, London, United Kingdom; c Universidad de Cartagena, Cartagena, Colombia; d Universidad de la Costa – CUC, Barranquilla, Colombia; e Mutual Ser, Cartagena, Colombia; f Corporación Universitaria Rafael Núñez, Cartagena, Colombia; g Universidade de São Paulo, São Paulo, Brazil; h University of Oxford, Oxford, United Kingdom; i Universidad Nacional de Colombia, Bogotá, Colombia

**Keywords:** Vaccine effectiveness, Covid-19, Mortality, Vaccines

## Abstract

**Background:**

In February 2021, Colombia began mass vaccination against COVID-19 using mainly BNT162b2 and CoronaVac vaccines. We aimed to estimate vaccine effectiveness (VE) to prevent COVID-19 symptomatic cases, hospitalization, critical care admission, and deaths in a cohort of 796,072 insured subjects older than 40 years in northern Colombia, a setting with a high SARS-CoV-2 transmission.

**Methods:**

We identified individuals vaccinated between March 1st of 2021 and August 15th of 2021. We included symptomatic cases, hospitalizations, critical care admissions, and deaths in patients with confirmed COVID-19 as main outcomes. We calculated VE for each outcome from the hazard ratio in Cox proportionally hazards regressions (adjusted by age, sex, place of residence, diabetes, human immunodeficiency virus, cancer, hypertension, tuberculosis, neurological diseases, and chronic renal disease), with 95% confidence intervals (CI).

**Findings:**

A total of 719,735 insured participants of 40 and more years were followed. We found 21,545 laboratory-confirmed symptomatic COVID-19 among unvaccinated population, along with 2874 hospitalizations, 1061 critical care admissions, and 1329 deaths, for a rate of 207.2 per million person-days, 27.1 per million person-days, 10.0 per million person-days, and 12.5 per million person-days, respectively. We found CoronaVac was not effective for any outcome in subjects above 80 years old; but for people 40-79 years of age, we found two doses of CoronaVac reduced hospitalization (33.1%; 95% CI, 14.5–47.7), critical care admission (47.2%; 95% CI, 18.5–65.8), and death (55.7%; 95% CI, 32.5–70.0). We found BNT162b2 was effective for all outcomes in the entire population of subjects above 40 years of age, significantly declining for subjects ≥80 years.

**Interpretation:**

Two doses of either CoronaVac in population between 40 and 79 years of age, or BNT162b2 among vaccinated above 40 years old significantly reduced deaths of confirmed COVID-19 in a cohort of individuals from Colombia. Vaccine effectiveness for CoronaVac and BNT162b2 declined with increasing age.

**Funding:**

UK National Institute for Health Research, the European Union's Horizon 2020 research and innovation programme, and the Bill & Melinda Gates Foundation.

**Research in context**

**Evidence before this study**

We systematically reviewed the evidence using a MEDLINE search through PubMed, with the combination of keywords “trial” AND (“coronavac” OR “sinovac” OR “pfizer” OR “bnt162b2”) AND (“vaccine” OR “vaccination”) AND (“covid” OR “coronavirus” OR “SARS-CoV-2”). In addition, we made a Google search, and used the Johns Hopkins’ International Vaccine Access Center systematic review of all studies reporting the effectiveness of COVID-19 vaccines. We included Phase 3 clinical trials in humans, published up to July 18th of 2021. We found six Phase 3 trials, one for CoronaVac (efficacy for symptomatic case of 83·5%; 95% confidence interval [CI], 65·4–92·1), and two for BNT162b2 (efficacies for symptomatic cases at all ages of 95·0 [95% CI, 90·3 to 97·6]; and 100·0% [95% CI, 75·3 to 100·0] in adolescents). For CoronaVac, there were two studies reporting effectiveness: one cohort study in Chile and a test-negative matched case-control design in Brazil. Both these latter studies show significant effectiveness to prevent death with CoronaVac vaccination.

**Added value of this study**

We report a significant reduction of death, critical care admission, and hospitalization for COVID-19, 14 days after the second dose of CoronaVac or BNT162b2 in a population 40–79 years old in Colombia, where at the time of this study, the Mu variant of interest was predominant. However, effectiveness against cases was negative for CoronaVac.

**Implications of all the available evidence**

BNT162b2 showed greater reductions than CoronaVac in death, critical care admission, and hospitalization for COVID-19 disease. Strengthening and increasing the speed of the immunization rollout with these vaccines may save lives in countries worldwide.

Alt-text: Unlabelled box

## Introduction

The SARS-CoV-2 virus first appeared in Wuhan, China in late-2019, causing a cluster of cases of COVID-19 that quickly disseminated worldwide to become the first large pandemic of the 21st century.^1^ As of May 17th, 2022, COVID-19 has killed at least 6·3 million people worldwide.^1^

Several efficacious and safe vaccines have been shown to prevent adverse outcomes due to COVID-19. The BNT162b2 vaccine has a reported efficacy of 95% (95% confidence interval [CI], 90.3–97.6) to prevent symptomatic cases,^2^ while the mRNA-1273 vaccine has reported 94% (95% CI, 89·3–96·8) efficacy for this outcome.^3^

CoronaVac has seen less peer-reviewed scrutiny of its efficacy, effectiveness, and safety, yet has been widely deployed in 72 countries by January 28th of 2022. Studies for CoronaVac have shown large heterogeneity in vaccine effectiveness. A recent clinical trial in Turkey showed CoronaVac had an efficacy to prevent symptomatic cases of 83·5% (95% CI 65·4–92·1), whilst a trial in Brazil showed a 51% vaccine efficacy against cases (95% CI, 36–62). A matched negative-control case-control study showed CoronaVac had an effectiveness of 47% to prevent COVID-19 cases, and 61% to prevent COVID-19 deaths in Brazil.^4^ Data from Chile show CoronaVac has an effectiveness of 66% to prevent cases.^5^ Based on this data, the Strategic Advisory Group of Experts on Immunization of the World Health Organization approved CoronaVac for emergency use in countries worldwide.^6^

Colombia started vaccinating healthcare workers in early February 2021, mainly using the BNT162b2 vaccine. The CoronaVac vaccine was then used to vaccinate people older than 80 years, and later expanded to those older than 60 years.^7^ After prioritization by occupations with higher risk of exposure, older age, and comorbidities, the vaccine was freely available in the country to the entire population without out-of-pocket expenses. As of 15th of August of 2021, overall vaccination coverage in Colombia with either vaccine was 40·8% for first doses and 27·1% for fully vaccinated individuals.

Given the need for real-world evidence on CoronaVac effectiveness, we consolidated data sources from one of the largest healthcare insurers in Colombia to provide evidence of the effectiveness of the vaccine in this setting. The present analysis provides a unique opportunity to assess vaccine effectiveness of CoronaVac alongside BNT162b2 COVID-19 vaccine in a setting where the Mu variant predominated in 2021. We aim here to assess the effectiveness of CoronaVac and BNT162b2 to prevent symptomatic cases, hospitalizations, critical care admissions, and deaths in patients with COVID-19 in Colombia.

## Methods

### Study design

We designed a retrospective cohort study to evaluate the effectiveness of the CoronaVac and BNT162b2 vaccination in Colombia between March 1st and August 15th of 2021. We only included insured subjects older than 40 years (Table 1) and excluded subjects with confirmed previous SARS-CoV-2 infection.

**Table 1:** Baseline characteristics of people vaccinated with two doses of CoronaVac and BNT162b2, and unvaccinated matched controls.

Parameters	Entire cohort	Unvaccinated	Two-dose CoronaVac	Two-dose BNT162b2
	*n* *=* *719735 (%)*	*n* *=* *539010 (%)*	*n* *=* *76729 (%)*	*n* *=* *56140 (%)*
**Age (yrs), *n* (%)**				
40–49	213468 (29.7)	197166 (36.6)	1503 (2.0)	5086 (9.1)
50–59	215482 (29.9)	166090 (30.8)	13422 (17.5)	21073 (37.5)
60–69	125601 (17.5)	73470 (13.6)	19011 (24.8)	20355 (36.3)
70–79	79877 (11.1)	41849 (7.8)	22788 (29.7)	8373 (14.9)
80+	85307 (11.9)	60435 (11.2)	20005 (26.1)	1253 (2.2)
**Sex, *n* (%)**				
Female	369599 (51.4)	272186 (50.5)	40004 (52.1)	31024 (55.3)
Male	350136 (48.6)	266824 (49.5)	36725 (47.9)	25116 (44.7)
**Type of municipality, *n* (%)**				
Other cities	475075 (66.0)	346473 (64.3)	58575 (76.3)	38188 (68.0)
Capital city	244660 (34.0)	192537 (35.7)	18154 (23.7)	17952 (32.0)
**Comorbidity, *n* (%)**				
Diabetes	42556 (5.9)	25265 (4.7)	7795 (10.2)	5591 (10.0)
HIV	2404 (0.3)	1851 (0.3)	136 (0.2)	232 (0.4)
Hypertension	95893 (13.3)	53722 (10.0)	21182 (27.6)	12106 (21.6)
Cancer	9308 (1.3)	5870 (1.1)	1679 (2.2)	968 (1.7)
Tuberculosis	110 (<0.1)	89 (<0.1)	6 (<0.1)	8 (<0.1)

This study was approved by the ethics committee of Universidad del Sinú, Cartagena, Colombia.

### Participants and data sources

**Figure 1:**
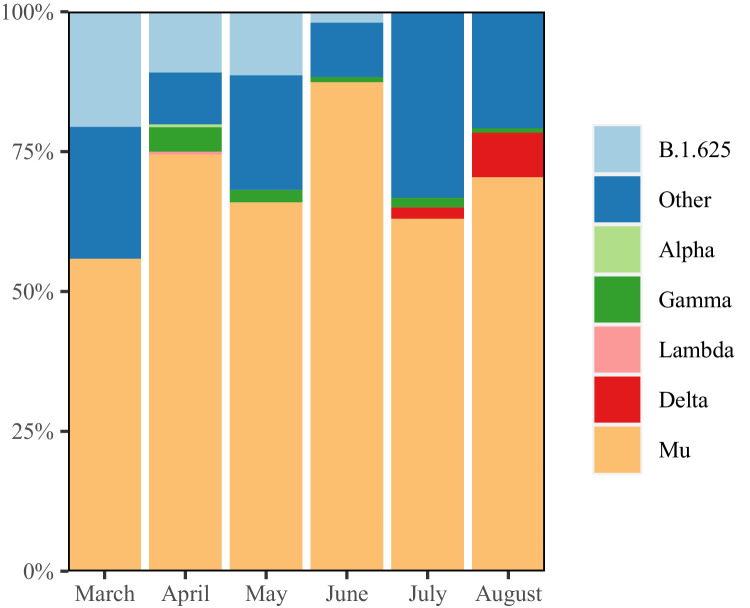
Monthly distribution of identified variants of SARS-CoV-2 in Colombia during the study period. Note: This is the monthly distribution of variants in the region of the study period in Colombia, with 779 samples analyzed by the Colombian National Institute of Health.^12^ These data are openly accessible. Samples without identified variants were excluded.

Our study population is the entire population enrolled in Mutual Ser, a health insurer of around 2·15 million people from Colombia. The insured are mostly low- and middle-income populations whose healthcare claims are subsidized by the government. The healthcare system in Colombia, includes healthcare provision for poor population (subsidized regime), for people who work (contributive regime), for military, teachers, and other populations (special regime), and for those who are willing to pay for private attention (out-of-pocket or private expenditure). Mutual Ser provides comprehensive health-related services under the subsidize regime for 25% in population of the Colombian departments of Atlántico, Bolívar, Cordoba, Magdalena, and Sucre. In 779 samples analyzed by the Colombian National Institute of Health, the Mu variant was predominant during this time-period (Figure 1). The data on variant frequency are publicly available.^8^

We identified comorbidities in insured patients (diabetes, human immunodeficiency virus (HIV), cancer, hypertension, tuberculosis, neurological diseases, and chronic renal diseases). To classify the diseases, we searched for diagnostic codes using the 10th version of the International Classification of Diseases (Supplementary Table S1), in the diagnosis of healthcare attentions in the insurer. We also used special health programs within the insurer to detect people with HIV, diabetes, hypertension, or cancer.

All persons with respiratory symptoms presenting to care were tested for COVID-19, as well as their contacts. The contact tracing algorithm searched for home family members and tested them, with or without symptoms.

### Vaccination status and outcome assessment

The second dose of CoronaVac is given 28 days after the first dose, while the second dose of BNT162b2 is scheduled 21 days after the first dose.

We extracted records of vaccination receipt from a national dataset of vaccinated individuals in Colombia covering the period March 1st to August 15th of 202.^9^^,^^10^ Vaccination records are collected by healthcare personnel administering the vaccine in health centers across the country, which enter them into a national official dataset of vaccinations. The records of the national dataset of vaccinations may be delayed therefore we collected the records of vaccinations two months after the end of the follow up period.

We defined four outcomes among persons with laboratory confirmed COVID-19: symptomatic illness, hospitalization, critical care admission and death, and extracted the dates of symptoms start from epidemiological records collected by Mutual Ser. Confirmatory laboratory testing comprised polymerase chain reaction (PCR), antigen, or IgM positive test. Incident cases were identified from both active and passive surveillance for COVID-19 symptoms (i.e., patients with cough, fever, difficult breathing, sore throat, or fatigue during the last past five days). Active surveillance was used by the insurer to trace and follow up contacts of laboratory-confirmed, symptomatic COVID-19 cases until free of symptoms. Hospitalizations and critical care admissions were collected from a dataset of all COVID-19 related healthcare attendances through a database collected by the insurer.

### Person-times of follow-up

The follow-up started on March 1st of 2021 for all patients. All patients were unvaccinated at this date, and vaccinated patients before this date were excluded. Once a subject was vaccinated (14 days after the first dose), this person changed status to vaccinated with the first dose of either BNT162b2 or CoronaVac. Then, if the person was vaccinated with two doses (14 days after the second dose), the person changed status to fully vaccinated. We only assessed the effectiveness of the second dose. This means a person can contribute to person-times as unvaccinated or fully vaccinated, depending on the dates of the second dose of each vaccine.

Person-times ended on the start of symptoms, when the patient had a positive symptomatic SARS-CoV-2 test, and when the patient was hospitalized, admitted to critical care, or died. These subjects were censured if these outcomes did not present at the end of follow-up on August 15th, 2021.

### Statistical analysis

We described the time to event for each outcome for cohorts using Kaplan–Meier curves. We fitted a Cox regression model with a vaccination status modeled as time-varying, to estimate the hazard ratios (HR) with 95% confidence intervals (CI). We tested the proportional hazards assumption using log-log plots. Vaccine effectiveness was calculated as one minus the HR. The analyses were reported crude and adjusted (by age, comorbidity, sex, and municipality). We also performed analyses to assess the potential differences of effectiveness depending on calendar time. For this assessment, we split the cohort into two analyses, one from March 1st until May 9th of 2021, and another cohort from May 10th to August 15th of 2021. These dates were chosen by diving the total study time of the main analysis into two halves. Both these cohorts had person-times depending on their start and end dates. The vaccine effectiveness in the split cohort was estimated similarly to the main analysis.

We stratified analyses by age (40–79, 80 years old or more), sex, and by presence or absence of comorbidities (diabetes, human immunodeficiency virus, cancer, hypertension, tuberculosis, neurological diseases, or chronic renal diseases), adding an interaction between vaccine doses and the strata (older age, sex, and comorbidity) to estimate the *p*-value of the difference between vaccine effectiveness in each stratum.

A *p*-value <0.05 was considered statistically significant for all analyses. All analyses were made in R (version 4.1.1), using the package ‘survival’ (version 3.-211).

### Role of the funding source

The funders of the study had no role in study design, data collection, data analysis, data interpretation, or writing of the report.

## Results

### Population

A total of 719,735 insured participants of 40 and more years were part of the cohort followed up from 1st of March of 2021 to August 15th of 2021, with a median age of 56 years old (IQR, 48––68), and 48·6% males (Table 1). The vaccine coverage for the first dose of all vaccines in the cohort was 25·1% at the end of follow-up.

The frequency of comorbidities is listed in Table 1, where hypertension was the most frequent comorbid disease (*n* = 95,893; 13·3%), followed by diabetes (*n* = 42,556; 5·9%).

We found 21,545 laboratory-confirmed symptomatic COVID-19 among unvaccinated population, along with 2874 hospitalizations, 1061 critical care admissions, and 1329 deaths, for a rate of 207·2 per million person-days, 27·1 per million person-days, 10·0 per million person-days, and 12·5 per million person-days, respectively.

### Effectiveness of CoronaVac

The rate of disease among the two-dose cohort of CoronaVac was 205·1 per million person-days for symptomatic cases, 28·4 per million person-days for hospitalizations, 9·3 per million person-days for critical care admissions, and 13·6 per million person-days for deaths.

The effectiveness of CoronaVac was significantly different according to age and the presence of comorbidities. We found CoronaVac was not effective for any outcome in the totality of subjects above 40 years old; but for people younger than 80 years of age, we found two doses of CoronaVac reduced hospitalization (33·1%; 95% CI, 14·5–47·7), critical care admission (47·2%; 95% CI, 18·5–65·8), and death (55·7%; 95% CI, 32·5–70·0). See Tables 2 and 3. In our analyses, subjects with comorbidities had significantly more CoronaVac effectiveness than subjects without these comorbid diseases (COVID-19 deaths were reduced among vaccinated with comorbidities by 44·6%; 95% CI, 20·6 to 61·3). Males had less CoronaVac effectiveness for COVID-19 symptomatic case and hospitalization (Table 3), without significant changes in the reduction of critical care admission or death.

**Table 2:** Effectiveness of two-dose vaccination with CoronaVac and BNT162b2 against Covid-19 cases, hospitalizations, critical care admissions, and deaths in Colombia.

Outcome and period	Laboratory-confirmed symptomatic Covid-19	Non-confirmed symptomatic Covid-19 illness
	Effectiveness (95% CI)*	Effectiveness (95% CI)*
**CoronaVac vaccination**		
Symptomatic case	-44.0 (-54.1 to -34.6)	-45.1 (-53.6 to -37.1)
Hospitalization	3.3 (-15.1 to 18.7)	-3.0 (-18.0 to 10.1)
Critical care admission	18.0 (-10.6 to 39.2)	13.6 (-13.2 to 34.0)
Death	21.4 (-0.7 to 38.6)	20.6 (-0.5 to 37.3)
**BNT162b2 vaccination**		
Symptomatic case	29.6 (21.1 to 37.2)	13.0 (5.3 to 20.0)
Hospitalization	54.2 (34.6 to 67.9)	45.5 (29.4 to 58.0)
Critical care admission	82.1 (56.5 to 92.6)	82.2 (60.1 to 92.1)
Death	93.5 (73.9 to 98.4)	94.1 (76.4 to 98.5)

**Note**: * Vaccine effectiveness (95% confidence intervals).

**Table 3:** Effectiveness of CoronaVac and BNT162b2 to prevent laboratory-confirmed symptomatic cases, hospitalizations, critical care, and deaths in Colombia, 14 days after the second dose, according to several scenarios.

Scenarios	Symptomatic case	Hospitalization	Critical care admission	Death
	Effectiveness (95% CI)*	Effectiveness (95% CI)*	Effectiveness (95% CI)*	Effectiveness (95% CI)*
**CoronaVac**				

**Age (yrs)**				
40–79	-22.1 (-32.8 to -12.3)	33.1 (14.5 to 47.7)	47.2 (18.5 to 65.8)	55.7 (32.5 to 71.0)
80+	-149.2 (-185.2 to -117.8) (<0.001)	-76.5 (-133.3 to -33.5) (<0.001)	-50.8 (-141.5 to 5.9) (<0.001)	-19.3 (-66.2 to 14.3) (<0.001)
**Sex**				
Female	-36.6 (-49.7 to -24.7)	20.8 (-3.8 to 39.5)	32.9 (-9.3 to 58.8)	35.2 (4.2 to 56.2)
Mal**e**	-51.4 (-67.3 to -37.0) (<0.001)	-12.5 (-41.4 to 10.5) (<0.001)	6.4 (-37.0 to 36.1) (0.080)	9.0 (-25.5 to 34.1) (0.072)
**Comorbidities**				
Without comorbidities	-74.6 (-92.2 to -58.7)	-23.6 (-59.7 to 4.3)	-13.4 (-69.4 to 24.1)	-17.1 (-64.4 to 16.6)
With comorbidities	-21.3 (-33.4 to -10.2) (<0.001)	19.3 (-2.2 to 36.3) (0.005)	36.7 (1.0 to 59.5) (0.002)	44.6 (20.6 to 61.3) (<0.001)

**BNT162b2**				

**Age (yrs)**				
40–79	32.2 (23.9 to 39.7)	59.7 (41.4 to 72.3)	85.9 (62.0 to 94.7)	96.7 (76.6 to 99.5)
80+	-48.5 (-178.7 to 20.8) (0.032)	-69.0 (-434.3 to 46.5) (0.019)	-30.0 (-850.6 to 82.2) (0.031)	34.0 (-374.8 to 90.8) (0.024)
**Sex**				
Female	31.2 (20.2 to 40.7)	66.0 (40.8 to 80.5)	87.1 (47.7 to 96.8)	93.9 (56.2 to 99.1)
Mal**e**	28.2 (14.0 to 40.0) (0.189)	40.6 (5.5 to 62.7) (0.183)	76.5 (26.0 to 92.5) (0.667)	93.2 (51.1 to 99.0) (0.991)
**Comorbidities**				
Without comorbidities	30.3 (17.5 to 41.2)	72.5 (44.6 to 86.4)	71.6 (22.6 to 89.6)	N.E.
With comorbidities	28.4 (16.1 to 38.8) (0.909)	40.7 (9.8 to 61.0) (0.128)	78.3 (47.2 to 91.1) (0.368)	87.2 (48.4 to 96.8) (0.980)

**Note**: * Vaccine effectiveness (95% confidence intervals) (*p*-value of the difference between the strata). N.E.: Not estimable.

In the analysis splitting the cohort to assess the effects of calendar time, CoronaVac reduced confirmed COVID-19 deaths in the first half of study-time by 17·2% (95% CI, -90·2 to 63·9) and 20·1% (95% CI, -8·8 to 41·30) in the second half. All the remaining outcomes were also non-significant in both cohorts split by half the calendar time of total study time.

### Effectiveness of BNT162b2

The rate of disease among the two-dose cohort of CoronaVac was 108·4 per million person-days for symptomatic cases, 10·8 per million person-days for hospitalizations, 1·7 per million person-days for critical care admissions, and 0·7 per million person-days for deaths.

We found BNT162b2 was effective for all outcomes in the entire population of subjects above 40 years of age (Table 2). We found that BNT162b2 effectiveness was different according to age, with older population having less reduction of all outcomes, while all other data stratification (comorbidities or not, and sex) did show significant differences and vaccination effectiveness when using two doses of BNT162b2 (Table 3).

The outcome with the highest BNT162b2 vaccine effectiveness with two doses in the entire population was death (93.5%; 95% CI, 73·9–98·4) (Table 2).

In the analysis stratifying the cohort by calendar time, the effectiveness of BNT162b2 was inestimable in the Cox proportionally hazards regression for COVID-19 deaths and critical care admissions. For COVID-19 confirmed symptomatic case, the effectiveness was 11·1% (95% CI, -532·2 to 87·5) in the first half and 43·3% (95% CI, 27·3 to 55·8) in the second half.

## Discussion

We found a COVID-19 mortality risk reduction of 93·5% (95% CI, 73·9 to 98·4) for those with two doses of BNT162b and 55·7% (95% CI, 32·5 to 71·0) among those between 40 and 79 years of age vaccinated with two doses of CoronaVac. The effectiveness of CoronaVac and BNT162b2 estimated in the present study must be put into the context of the population studied. Our cohort of CoronaVac vaccinees is older than previous evaluations, with a median age of 68 years. This may bias towards lower effectiveness, especially for CoronaVac, which is reported to have lower effectiveness in older ages.^11^ Our evaluation of the effectiveness of BNT162b2 is in line with previous studies showing a strong effectiveness for this vaccine.

Our results would also need to be put into the context of high pre-existing SARS-CoV-2 immunity through previous natural infection in the region of the study. In serosurveys prior to the start of the present analysis (in late-November of 2020), a high prevalence of antibodies against SARS-CoV-2 was found in two of the capital cities in this region, with a 59% seroprevalence in Monteria (capital of Cordoba) and 53% in Barranquilla (capital of Atlántico).^12^ The extent of COVID-19 community transmission and pre-existing immunity acquired prior to the initiation of the vaccine program is likely to have reduced the power of this study, and may have decreased the incremental estimates of effectiveness of the vaccines evaluated.

Seventy-two countries worldwide currently approve CoronaVac for emergency use (on May 19th of 2022),^13^ representing most of the world population. Our study contributes to the increasing literature showing CoronaVac is effective to prevent severe adverse outcomes of Covid-19. A previous study from Arregocés-Castillo et al.^14^ in Colombia assessed the effectiveness of COVID-19 vaccines against hospitalization, death after hospitalization, and death without hospitalization in the entire population older than 60 years in the country. They found BNT162b2 reduced death after hospitalization by 94·8% (95% CI, 93·3 to 96·0) in people older than 60 years and 92·7% (95% IC, 85·4 to 96·4); while the CoronaVac effectiveness for this outcome was 72·1% (95% CI, 70·1 to 73·9) in those older than 60 years and 66·3% (95% CI, 63·4 to 69·0) in people over 80 years old. Our study uses data from poor population in northern Colombia, which is different from the Arregocés-Castillo et al. study that used the entire Colombian population. Our results for CoronaVac reported, descriptively, a lower effectiveness than the previous study in the entire Colombian population, while for BNT162b2 we found a higher effectiveness. The causes of these differences are unknown, but several hypotheses are worth exploring. The sample of our study was composed of people with low socioeconomical status, which is a driver of infection and adverse outcomes. Studies from United States,^15^^,^^16^ Germany,^17^ UK,^18^ Chile,^19^ and Colombia^20^ have shown population with lower socioeconomic status have higher infection rate,^16^, ^17^, ^18^^,^^20^^,^^21^ suggesting our population may have increased infection dynamics and more seroprevalence at the time of the study. A Colombian study^20^ showed that during the pandemic, lower socioeconomic status was associated to longer time at work when symptomatic, longer time at work with a known positive contact, longer time working outside home, and longer time between symptoms and test date and test result. A study in the UK also showed how different infection waves were associated to different risks of infection according to socioeconomic status. This increased infection susceptibility could have potentially increase previous immunity among poor population, potentially decreasing the effectiveness of vaccination of CoronaVac in our sample. Another potential cause for these differences is lower statistical power in our sample, with Arregocés-Castillo et al. studying 1.4 million subjects in the unvaccinated cohort. Another difference between our analysis and the previous study is that the Arregocés-Castillo et al. assessment stratified death into two outcomes (with and without hospitalization), with lower effectiveness of death without hospitalization compared to deaths with hospitalizations. Our analyses do not stratify death into these categories, making the differences between studies less marked.

A study from Brazil reported a 50% reduction in symptomatic cases using a matched test-negative case-control study.^4^ Another large cohort in Brazil reported CoronaVac had a 73·7% effectiveness against COVID-19 deaths, 73·8% for critical care admission, and 52·7% for infection.^11^ In the present study, we did not find a significant reduction from receiving two doses of CoronaVac in symptomatic cases of confirmed COVID-19. However, we did find a significant reduction in deaths in younger population and more effectiveness for more severe outcomes, suggesting that CoronaVac may have greater efficacy against more severe disease. A report in Chile showed an effectiveness of 67% for symptomatic cases and 77·7% (95% CI, 25–100) for deaths with confirmed COVID-19.^5^ Chile vaccinated 90% of its population with CoronaVac,^5^ with a strong reduction of daily cases during April and May of 2021.^1^ Despite this, Chile reported the largest number of daily cases on June 10th of 2021 since the start of the pandemic.^1^ Chilean data might suggest that the effectiveness of CoronaVac is lower for symptomatic cases^4^ and waning overtime.^22^ The trends and peak of cases after 50% coverage with CoronaVac in Chile also might be explained by the reduced or no protection against infections or symptomatic cases in older population, as the present study shows. These hypotheses need further study.

Post-licensure studies of BNT162b2 have been published for Israel,^23^^,^^24^ United States,^25^^,^^26^ and the UK.^27^ The present study shows BNT162b2 has strong protection against symptomatic cases, hospitalization, critical care, and deaths. COVID-19 vaccination coverage of fully vaccinated people by June 10th of 2021 was 57% with BNT162b2 in Israel; and 43% in the UK, vaccinating with BNT162b2, Vaxzevria, mRNA-1273, and JNJ-78436735. The 7-day average number of deaths was two for Israel and eight for the UK on June 10th of 2021 (from a previous peak of 65 and 1248, respectively). The reduction of cases was also substantial in these two countries.

Colombia carries out genomic surveillance of SARS-CoV-2 and its variants, albeit with much less intensity than many high-income settings. According to the limited surveillance data available, the Mu variant predominated in the region in between March and August of 2021,^12^ with an estimated 60% prevalence of average monthly samples^12^ (Figure 1). The Mu variant has shown more resistance to SARS-CoV-2 antibodies than the Beta variant in a recent study.^28^ Previous studies show BNT162b2 has effectiveness against Alpha, Beta, and Gamma variants of SARS-CoV-2.^11^^,^^29^, ^30^, ^31^ Our study suggests that both BNT162b2 and CoronaVac offer protection against Covid-19 severe outcomes related to the Mu variant as well.

In contrast to BNT162b2, we found a significant increase in symptomatic COVID-19 cases occurring 14 days or more after of the second CoronaVac dose compared to unvaccinated subjected. For this outcome, we cannot eliminate the possibility of a health seeking bias that would increase the frequency of disease among vaccinated. This bias would only have an effect in milder outcomes, such as symptomatic and hospitalized COVID-19, and would be less likely to occur in more severe outcomes that are likely to universally result in health seeking behavior, such as death or critical care admission. Other bias affecting this outcome may be related to testing differences, although by selecting patients with less than ten days between testing and symptoms start did not change the direction of the results in this outcome.

The evaluation of interventions outside the controlled environment of a clinical trial setting is important, especially for mass interventions such as COVID-19 vaccination. Our study compiles and reports data from of a large insurer in Colombia and shows the effect of CoronaVac and BNT162b2 in the field. As with any observational study, ours has limitations and strengths. Our main limitation is related to potential health seeking biases, especially the non-hard outcomes, where the effectiveness was null or significatively negative (i.e., vaccination increased the disease rate for CoronaVac). Another limitation is the potential misclassification of COVID-19 outcomes due to delayed testing, although in our sensitivity analysis, the exclusion of those tested after ten days of symptoms start did not significantly alter the results. The data in the present study only had a mean follow-up of 107 days for CoronaVac, therefore we could not assess waning immunity. The frequency of some comorbidities is relatively low for the older population in present cohort. In the United States, the prevalence of controlled hypertension was 49% in 2015 in the population over 60 years old,^32^ and another study in Colombia reported a prevalence of treated hypertension of 40%.^33^ One potential cause for these differences is underreporting. Another possible explanation is that our sample comes from low-income backgrounds, therefore potentially increasing the number of subjects with undetected comorbidities. Our results have to be interpreted in the light of these limitations.

Taking the entire evidence into context, the data suggest the effectiveness of two doses of CoronaVac and BNT162b2 to prevent COVID-19 deaths could be substantial in a scenario where Mu predominates. More studies are needed to assess its protection against milder and asymptomatic disease, against other predominant variants, against older population for CoronaVac, and the potential vaccine waning of its effectiveness.

## Contributors

Dr. Paternina-Caicedo conceptualized the analysis, wrote the original draft, reviewed, and edited the manuscript, visualized, and made the formal analysis. Dr. Jit aided the methodology, reviewed, and edited the manuscript. Dr. Alvis-Guzman conceptualized the study, contributed to methodology, supervised the research, and reviewed the manuscript. Dr. Fernandez, Mr. Hernandez, and Dr. Paz aided conceptualizing, data curation, project administration, and contributed reviewing the drafts of the manuscript. Dr. Rojas-Suarez, Dr. Dueñas, and Mr. Alvis-Zakzuk contributed to validation of results, methodology, and reviewing the drafts of the manuscript. Dr. Smith contributed to the design, aided the methodology, and reviewed the drafts of the manuscript. Dr. De la Hoz-Restrepo conceptualized, contributed to the methodology, supervised, and review and edited the draft of the manuscript. All authors reviewed and approved the final manuscript for submission. Dr. Paternina-Caicedo, Dr. Fernandez, and Mr. Hernandez accessed and verified the original data for analysis.

## Data sharing statement

Data for this paper will be shared on a reasonable request without identifiers after receiving a signed data-sharing agreement where the researching requesting the data commits, among other things, to the use of the data for research proposes, not to identify any individual, and destroy the data after all analysis are completed.

## Declaration of interests

No author declares any conflicts of interest.

## Appendix Supplementary materials

Image, application 1

Image, video 2

Image, video 3

Image, video 4
