# Supplementary material for: Effectiveness of CoronaVac and BNT162b2 COVID-19 mass vaccination in Colombia: A population-based cohort study
Source: Lancet Reg Health Am. 2022 Jul 1;12:100296. doi: 10.1016/j.lana.2022.100296 (PMC9246705; doi:10.1016/j.lana.2022.100296)
Supplement: Supplementary file 1 [file mmc1.docx]

***Editorial disclaimer:*** *This translation in Spanish was submitted by the authors and we reproduce it as supplied. It has not been peer reviewed. Our editorial processes have only been applied to the original abstract in English, which should serve as reference for this manuscript.*

**Resumen**

**Antecedentes**: En febrero de 2021, Colombia inició la vacunación masiva contra el Covid-19 utilizando principalmente vacunas BNT162b2 y CoronaVac. Nuestro objetivo fue estimar la efectividad de la vacuna (EV) para prevenir casos sintomáticos, hospitalización, ingreso en cuidados críticos y muertes por Covid-19 en una cohorte de 796 072 asegurados mayores de 40 años en el norte de Colombia, en un escenario con alta transmisibilidad por SARS-CoV-2.

**Metodología**: Identificamos a las personas vacunadas entre el 1 de marzo de 2021 y el 15 de agosto de 2021. Incluimos casos sintomáticos, hospitalizaciones, admisiones en cuidados intensivos y muertes en pacientes con covid-19 confirmado como resultados principales. Calculamos la EV para cada resultado a partir del cociente de riesgos instantáneos en regresiones de riesgos proporcionales de Cox (ajustado por edad, sexo, lugar de residencia, diabetes, virus de la inmunodeficiencia humana, cáncer, hipertensión, tuberculosis, enfermedades neurológicas y enfermedad renal crónica), con un 95% intervalos de confianza (IC).

**Hallazgos**: Se siguieron un total de 719.735 participantes asegurados de 40 y más años. Encontramos 21.545 covid-19 sintomáticos confirmados por laboratorio entre la población no vacunada, junto con 2.874 hospitalizaciones, 1.061 admisiones en cuidados intensivos y 1.329 muertes, para una tasa de 207,2 por millón de días-persona, 27,1 por millón de días-persona, 10,0 por millón de personas -días y 12,5 por millón de días-persona, respectivamente. Encontramos que CoronaVac no fue efectivo para ningún resultado en la los sujetos mayores de 90 años; pero para las personas de 40-79 años, encontramos que dos dosis de CoronaVac redujeron la hospitalización (33,1 %; IC del 95 %, 14,5 a 47,7), la admisión a cuidados intensivos (47,2 %; IC del 95 %, 18,5 a 65,8) y la muerte (55,7%; IC 95%, 32,5-70,0). Encontramos que BNT162b2 fue efectivo para todos los resultados en toda la población de sujetos mayores de 40 años, disminuyendo significativamente para personas de más de 80 años.

**Interpretación**: Dos dosis de CoronaVac en población entre 40 y 79 años, o BNT162b2 entre vacunados mayores de 40 años redujeron significativamente las muertes por Covid-19 confirmado en una cohorte de individuos de Colombia. La efectividad de las vacunas CoronaVac y BNT162b2 disminuyeron con el aumento de la edad.
