# Supplementary material for: Effectiveness of CoronaVac and BNT162b2 COVID-19 mass vaccination in Colombia: A population-based cohort study
Source: Lancet Reg Health Am. 2022 Jul 1;12:100296. doi: 10.1016/j.lana.2022.100296 (PMC9246705; doi:10.1016/j.lana.2022.100296)
Supplement: Supplementary file 2 [file mmc2.docx]

**Effectiveness of CoronaVac and** **BNT162b2** **Covid-19 Mass Vaccination in Colombia: a population-based cohort study**

Angel Paternina-Caicedo, MSc^a,*^; Mark Jit, PhD^b^; Nelson Alvis-Guzmán, PhD^c,d^; Juan Carlos Fernández, MSc, MBA^e^; José Hernández, MSc^e^; Justo Jesus Paz-Wilches, MD^e^; José Rojas-Suarez, MSc^c^; Carmelo Dueñas-Castell, MD^c^; Nelson J. Alvis-Zakzuk, MSc ^d,f^; Adrian D. Smith, DPhil^g^; Fernando De La Hoz-Restrepo, PhD^h^

**Affiliations**

^a^ Universidad del Sinú, Cartagena, Colombia.

^b^ London School of Hygiene & Tropical Medicine, London, United Kingdom.

^c^ Universidad de Cartagena, Cartagena, Colombia.

^d^ Universidad de la Costa – CUC, Barranquilla, Colombia.

^e^ Mutual Ser, Cartagena, Colombia.

^f^ Universidade de São Paulo, São Paulo, Brazil.

^g^ University of Oxford, Oxford, United Kingdom.

^h^ Universidad Nacional de Colombia, Bogotá, Colombia.

***Corresponding author**: Angel Paternina-Caicedo, MD MSc. Address: Tv. 54 #41-117, Universidad del Sinú, Cartagena, Colombia. Email: [apaterninac@unisinucartagena.edu.co](mailto:apaterninac@unisinucartagena.edu.co), angelpaterninacaicedo@gmail.com. Phone: +57-316-3659567.

Table S1. Diagnostic codes for comorbidities.

| **Disease** | **Diagnostic code (10 version of the international classification of diseases)** |
| --- | --- |
| Cancer | C221, D093, D020, D059, D042, D043, D048, D044, D046, D041, D045, D049, D075, D072, D090, D071, D023, D067, D014, D076, D097, D013, D022, D010, D069, D060, D070, D002, D061, D015, D000, D092, D074, D012, D051, D050, D099, C961, C448, C068, C148, C950, C914, C910, C911, C919, C920, C921, C929, C925, C931, C933, C930, C913, C924, C959, C837, C830, C851, C833, C812, C811, C810, C819, C820, C821, C822, C829, C835, C843, C839, C836, C834, C832, C859, C844, C850, D038, D037, D031, C433, C439, C437, C436, C431, C435, C459, C945, C900, C887, C917, C927, D057, C845, C817, C827, C838, C857, D477, C902, D45X, C923, D34X, D24X, D233, D231, D239, D300, D170, D172, D173, D443, D440, D486, D411, D485, D381, D414, D420, D433, D487, D373, D374, D431, D432, D430, D376, D480, D383, D391, D410, D390, C759, C089, C024, C099, C241, C01X, C069, C250, C760, C020, C252, C412, C690, C693, C166, C039, C140, C300, C750, C07X, C753, C080, C749, C73X, C549, C320, C139, C751, C329, C029, C509, C741, C720, C119, C696, C109, C102, C672, C111, C763, C65X, C442, C443, C444, C440, C446, C441, C445, C447, C449, C58X, C501, C506, C61X, C692, C570, C19X, C680, C52X, C679, C519, C700, C709, C240, C349, C773, C770, C775, C772, C403, C401, C414, C410, C402, C409, C471, C962, C579, C577, C257, C481, C319, C269, C80X, C762, C183, C210, C163, C181, C062, C021, C340, C323, C716, C710, C180, C182, C186, C187, C184, C189, C211, C380, C505, C503, C504, C502, C539, C162, C251, C602, C170, C719, C530, C541, C632, C159, C152, C150, C169, C531, C543, C161, C779, C229, C411, C419, C172, C179, C260, C004, C000, C003, C005, C711, C343, C342, C714, C713, C341, C381, C383, C765, C764, C724, C723, C301, C699, C400, C56X, C051, C050, C059, C254, C259, C717, C609, C482, C500, C164, C049, C20X, C480, C64X, C313, C311, C312, C310, C729, C490, C495, C494, C492, C491, C493, C496, C499, C155, C621, C620, C629, C37X, C761, C55X, C715, C171, C791, C795, C798, C793, C787, C784, C781, C796, C786, C780, C790, C799, C809, C97X |
| Diabetes | E121, E120, E125, E124, E128, E123, E126, E129, E101, E100, E105, E107, E104, E108, E103, E102, E106, E109, E141, E145, E147, E144, E148, E143, E142, E146, E149, E111, E110, E115, E117, E114, E118, E113, E112, E116, E119, E131, E135, E138, E136, E139 |
| Neurological disease | F060, G824, G825, F002, F001, F000, F009, F020, F019, G20X, I631, G808, F789, F781, F788, F228, F068, G804, G800, G809, G821, F729, F720, F721, F709, F700, F701, F708, F719, F710, F711, F718, F731, F799, F790, F791, F798, F067 |
| Chronic renal disease | N181, N189, I120, I129, N171, N170, N179, N19X, N180, N178, N188 |
| Hypertension | I10X, I159, I120, I158 |
| Tuberculosis | A158, A150, A153, A151, A159, A169, A167 |
